# Critical need to assess modified and un-modified peptides in C-peptide standard materials

**DOI:** 10.1016/j.jmsacl.2022.07.003

**Published:** 2022-07-22

**Authors:** Zengru Wu, Kuanysh Kabytaev, Jianying Mu, Shawn Connolly, Nigel J. Clarke, Randie Little, Michael J. McPhaul

**Affiliations:** Quest Diagnostics Nichols Institute, San Juan Capistrano, CA, USA; Department of Pathology & Anatomical Sciences, School of Medicine, University of Missouri, Columbia, MO, USA; Quest Diagnostics Nichols Institute, San Juan Capistrano, CA, USA; Department of Pathology & Anatomical Sciences, School of Medicine, University of Missouri, Columbia, MO, USA; Quest Diagnostics Nichols Institute, San Juan Capistrano, CA, USA; Department of Pathology & Anatomical Sciences, School of Medicine, University of Missouri, Columbia, MO, USA; Quest Diagnostics Nichols Institute, San Juan Capistrano, CA, USA

**Keywords:** CRM, Certified Reference Material, IRB, Internal Review Board, LC-MS/MS, Liquid Chromatography-Tandem Mass Spectrometry, MS, mass spectrometry, NMIJ, National Metrology Institute of Japan, C-peptide, Standardization, Reference materials, The first paragraph is missing. Please add back.

C-peptide assays have been widely used as a measure of insulin secretion to assess pancreatic beta-cell function [1]. High levels of C-peptide may reflect insulin resistance, insulinoma, and kidney disease. A low level is usually present in patients with type 1 diabetes and, under certain circumstances, type 2 diabetes [2]. As a biomarker, C-peptide has several advantages over insulin: (i) the degradation rate of C-peptide in the body (20-30 minute half-life) is slower than that of insulin (3 to 5 minute half-life), which provides a more stable test window within a fluctuating beta cell response, (ii) C-peptide is cleared in the peripheral circulation at a constant rate, whereas insulin is cleared variably making direct measurement less consistent, and (iii) in insulin-treated diabetic patients, the measurement of C-peptide avoids cross-reactivity between exogenous and endogenous insulin.

We have previously developed and validated a high-throughput, quantitative, multiplexed liquid chromatography-tandem mass spectrometry (LC-MS/MS) assay for intact insulin and C-peptide [3]. C-peptide was enriched from patient sera using monoclonal antibodies immobilized on magnetic beads and processed on a robotic liquid handler. Eluted C-peptide was analyzed by LC-MS/MS.

Commercial C-peptide assays are still not standardized to ensure that test results are comparable among laboratories to provide unambiguous diagnosis and treatment monitoring [4]. Certified reference materials (CRM) are well-characterized, highly pure, non-matrix material that reside at the top of the traceability chain. In this study, we evaluated two CRMs for C-peptide: 6901-b from National Metrology Institute of Japan (NMIJ Lot# 058, 1.9% modified) and newly released C-161-0.1 ML from Cerilliant (Lot# FN04221901, 5.6% modified) to assess their utility and comparability. The CRM from NMIJ is a lyophilized powder and that from Cerilliant is a solution in a sealed ampule. The certified values from both sources include the proportion of unmodified C-peptide and modified C-peptide (deamidated C-peptide, and pyroglutamated C-peptide).

In this study 45 patient serum samples were analyzed. These specimens were originally collected for an Insulin/C-Peptide LC-MS/MS assay (Quest Diagnostics test code 93103). An institutional review board (IRB) waiver was obtained for collection and for utilization of residual clinical samples. The IRB sponsor protocol number is BR13-002 and the IRB protocol number is 20121940. Noticeable bias was found when comparing C-peptide LC-MS/MS results obtained using calibrators prepared using unmodified C-peptide versus total C-peptide. In contrast, the bias was reduced when mass concentrations of unmodified C-peptide were used to assign calibrator concentrations, regardless of the manufacturer (Fig. 1).

**Fig. 1:**
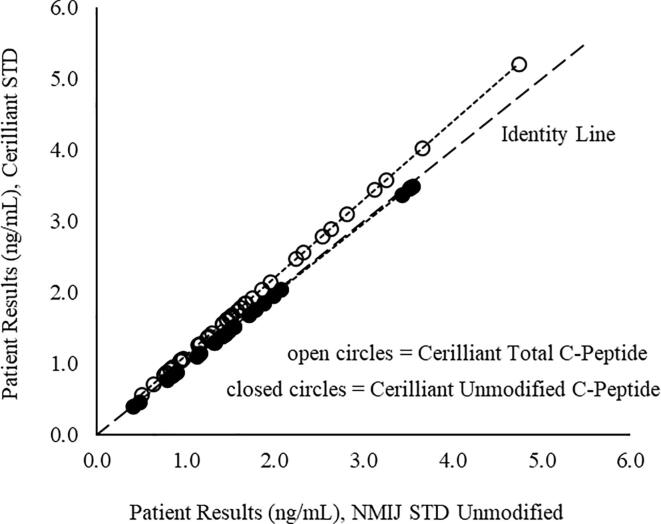
Correlation of C-peptide results for patient samples using different calibrator sources (n = 45).

These results reflect the ability of LC-MS/MS assays to differentiate between unmodified and modified C-peptide (e.g., pyroglutamylated forms). The N-terminal amino acid of C-peptide is glutamate, which can lose a water molecule to form pyroglutamate with a ring structure during its manufacturing process. The change in molecular weight will make it invisible in the mass spectrometric detection and it could change the retention time during HPLC separation. The degree of modification varies for different lots and manufacturers. The formation of pyroglutamate is spontaneous *in vivo* and *in vitro*, but the degree of formation can be controlled to within a certain range during clinical laboratory assessment by specimen stability data at various conditions. Using unmodified C-peptide, good agreement (slope = 0.94, intercept = 0.009, r^2^ = 0.97) was achieved in the interlaboratory comparison studies using patient serum samples (n = 39) with University of Missouri, which uses a CRM to calibrate their internal isotopically-labeled standard [4]. During routine operation, the lot-to-lot variation of modified C-peptide percentages should be taken into consideration.

We conclude that CRM of C-peptide from Cerilliant and from NMIJ behave identically when the mass concentration of unmodified C-peptide is used to assign calibrator concentrations. To avoid potential bias and to achieve comparable patient results among laboratories, the mass concentration of unmodified C-peptide (rather total C-peptide) in CRMs should be used when making calibrators for LC-MS/MS-based C-peptide assays. Our results suggest that variation in the relative amount of modified C-peptide forms can be an obstacle to standardization, especially when comparing MS-based assays with immunoassays, which are unable to discriminate between intact and modified C-peptide forms.

## Declaration of Competing Interest

The authors declare that they have no known competing financial interests or personal relationships that could have appeared to influence the work reported in this paper.
